# The development of the 'COVID-19 Psychological Resilience Model' and its efficacy during the COVID-19 pandemic in China

**DOI:** 10.7150/ijbs.50127

**Published:** 2020-08-27

**Authors:** Zongling He, Jiajia Chen, Keliang Pan, Yuchuan Yue, Teris Cheung, Yin Yuan, Na Du, Yan Zhao, Yusu Feng, Die Zhou, You Zhou, Fengmei Lu, Yong Chen, Manxi He, Yu-Tao Xiang

**Affiliations:** 1 The Clinical Hospital of Chengdu Brain Science Institute, School of Life Science and Technology, University of Electronic Science and Technology of China, Chengdu, 610054, PR China.; 2 The Mental Health center of Chengdu, Sichuan, 610036, China.; 3 School of Nursing, Hong Kong Polytechnic University, Hong Kong SAR, China.; 4 Unit of Psychiatry, Institute of Translational Medicine, Faculty of Health Sciences, University of Macau, Macao SAR, China.; 5 Center for Cognition and Brain Sciences, University of Macau, Macao SAR, China.

**Keywords:** novel coronavirus disease, live media, hotline consultation, video intervention, on-site crisis intervention

## Abstract

During the novel coronavirus disease 2019 (COVID-19) outbreak, traditional face-to-face psychological interventions have been suspended due to high risks of rapid transmission. Developing an effective online model of psychological intervention is deemed necessary to deal with the mental health challenges brought up by this disease. An integrated psychological intervention model coined 'COVID-19 Psychological Resilience Model' was developed in Chengdu, China including live media, 24-hour hotline consultations, online video intervention and on-site crisis intervention sessions to provide services to those in need. A total of 45 episodes of live media programs on COVID-19 outbreak-related psychological problems were broadcasted with over 10 million views. A total of 4,236 hotline consultations were completed. More than 50% of the clients had positive feedback about the hotline consultations. A total of 223 cases received online video intervention, of which 84.97% were redirected from the hotline consultation and 15.03% from COVID-19-designated hospital and community-based observation spots. Seventy one-on-one psychological interventions were conducted with 39 COVID-19 patients, and one-third were treated with medication. Additionally, 5 training sessions were conducted to 98 frontline medical staff. This 'COVID-19 Psychological Resilience Model' is proven effective to the general population during the COVID-19 pandemic. We have greatly improved the overall mental health of our target population during the COVID-19 pandemic. This model could provide valuable experiences and serve as a reference guide for other countries to offer effective psychological intervention, and reduce detrimental negative mental health outcomes in public health emergency.

## Introduction

The novel coronavirus disease 2019 (COVID-19) pandemic is a global public health crisis. After it was first reported in Wuhan, Hubei province in China at the end of December, 2019, COVID-19 was rapidly transmitted to all provinces across China and were also reported in more than 200 countries worldwide. This rapid transmission has posed a serious threat to human life. A range of unprecedented measures were adopted to contain the virus, including isolation of suspected and confirmed cases, social distancing, tracing and monitoring of close contacts and limiting the number of people in public gathering 1. The COVID-19 pandemic not only threatened human life but also imposed a serious impact on the society, economy and mental health. The outbreak itself and the mass quarantine control measures have led to common health problems such as fear, anxiety and panic, which may escalate into further negative psychological reactions, including adjustment disorder, anxiety disorder and depression 2-7. Patients with COVID-19, close contacts, quarantined cases, frontline medical staff, the general public and even health care professionals have been confronted with different levels of psychological stress 4, 8. The World Health Organization (WHO) recommended that a speedy assessment of identifying outbreak-associated psychological stressors is urgently needed 9. As such, mental health crisis interventions have been integrated into the overall deployment of disease prevention and treatment by the Central Health Authority of China and various national academic groups. The absence of mental health and psychosocial support systems increases the risks of psychological distress and progression to psychiatric disorders during crisis events 9-11. Therefore, there is a pressing need to establish a systematic and effective model of psychological intervention to address the mental health challenges caused by the COVID-19 outbreak.

**Figure 1:**
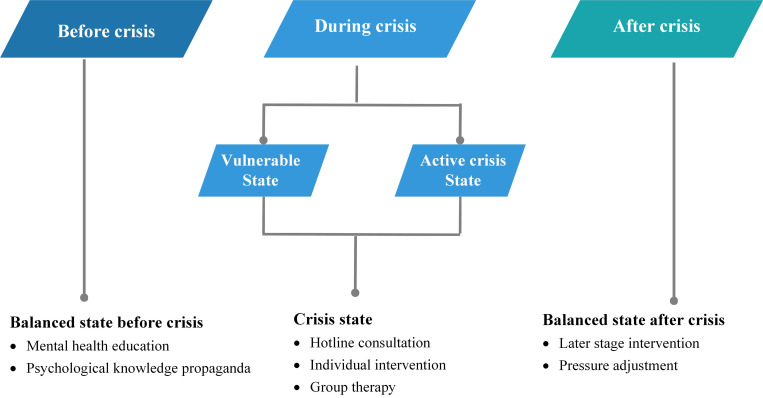
Different approaches to each psychological state during crisis events.

When confronted with crisis events, individuals may experience three psychological states. The focus and approach of interventions vary at different stages 12-14. Specifically, in the balanced state before a crisis, individuals apply coping skills and self-adjustment techniques to maintain a homeostasis between themselves and the environment. At this stage, it is appropriate to conduct psychological education to mentally prepare the individuals to deal with the crisis so as to reduce the negative outcome. In the crisis state (during the crisis), individuals start to experience emotional disturbance or even psychological breakdown due to extreme tension and anxiety. At this stage, intervention should include 24-hotline consultation, individual intervention and group therapy to help people overcome their mood instability. In the balanced state (after a crisis), individuals have gained experience, learned stress management/adjustment skills, and they could recover from the crisis or even surpass their pre-crisis level (**Figure 1**). Thus, timely and strategic psychological intervention in different stage is extremely critical to reduce undesirable mental health outcomes.

China has made tremendous progress in applying psychological interventions to successfully address the public psychological crisis after several disasters, for example, the 2008 Wenchuan earthquake 15. However, since COVID-19 has a very high risk of rapid human-to-human transmission, traditional face-to-face intervention is almost impossible 1, 16. In this light, the mode of psychological intervention delivery should differ from those commonly used in a natural disaster or a sudden public health crisis 5, 9, 17. Thus, we proposed that the design of the psychological intervention during the COVID-19 pandemic should be dynamic and adaptive to different stages of individuals' reaction to crisis events, i.e., a balanced state before and during the crisis. The key point is that during the pandemic, mental health professionals should actively participate in the overall intervention process so that mental health and psychosocial responses can be mobilized in a timely fashion. In this process, psychological intervention should include three simultaneous activities: (1) promoting mental health knowledge and improving the public's psychological preparation for crisis by means of live media; (2) offering user-friendly psychological assistance to relieve negative emotion by 24-hour hotlines and online videos; (3) managing cases with severe mental problems and providing on-site psychological crisis intervention. In this process, a psychological intervention called the COVID-19 Psychological Resilience Model was developed.

## Methods

This study was conducted ethically in accordance with the World Medical Association Declaration of Helsinki and the study protocol was approved by the research ethical committee of the Mental Health Center of Chengdu, China.

### Organization set up

Under the leadership of the government, the Mental Health Center of Chengdu, China, established a leading group which comprised of authoritative experts in crisis intervention, and responsible for the overall planning. According to the “Guideline for the Emergent Psychological Crisis Intervention during the Novel Coronavirus Pneumonia Pandemic” released by the National Health Commission of China on the 25^th^ of January 2020, this leading group developed working programs and manuals for psychological intervention based on various channels people were affected by the COVID-19 pandemic in China. The entire work flow was implemented in the following steps.

#### Step 1: Mobilization and Preparation

Initially, the leading group formulated work manuals for psychological intervention and conducted a series of pre-training workshops for the back-up team involved in the psychological intervention.

#### Step 2: Multidisciplinary Team Establishment

**Figure 2:**
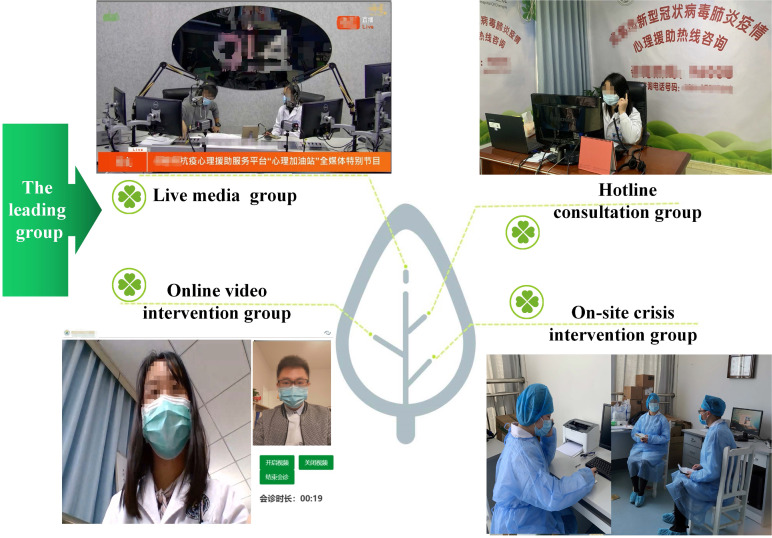
The organizational framework of the psychological intervention team for the COVID-19 pandemic.

A psychological intervention team was established supported by mental health professionals in the Mental Health Center of Chengdu, China. This team consisted of 26 multidisciplinary members, including psychiatrists, psychiatric nurses, psychotherapists and psychologists. Subsequently, they were divided into four groups: 1) live media group; 2) hotline consultation group; 3) online video intervention group and; 4) on-site crisis intervention group (**Figure 2**). Each group had a leader responsible for regularly reporting of their daily work progress to the leading group.

#### Step 3: Feedback Mechanism

Problem feedback mechanisms were established. The leading group listened to the feedback report of each team regularly so that they could supervise and adjust the intervention work in a timely and prompt manner.

### Structured psychological intervention program implementation

**Figure 3:**
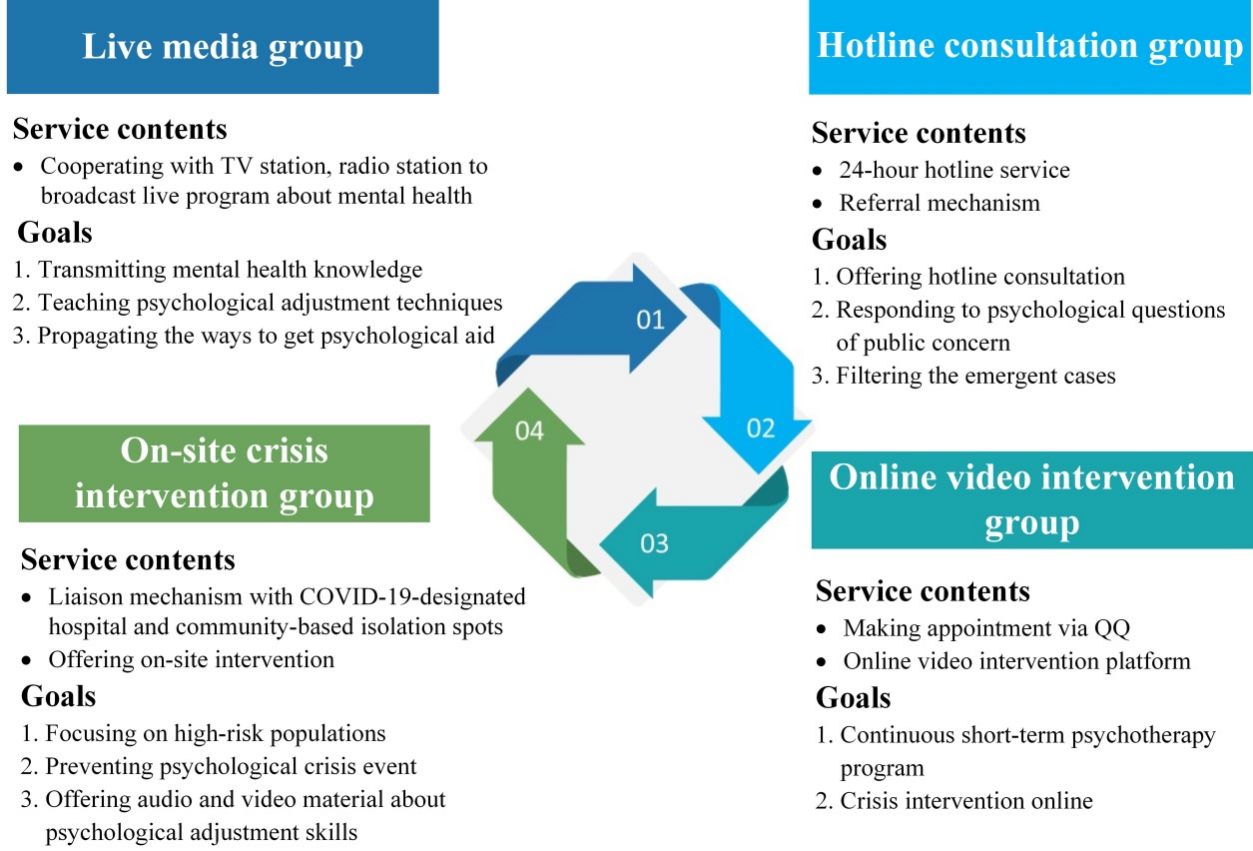
Structured framework of psychological intervention during the COVID-19 pandemic.

We established a 4-tiered structured framework of psychological intervention (**Figure 3**, **Table 1**). At the bottom of the framework (1^st^ tier) was mental health public awareness, arguably the most widespread layer covering a large number of lay persons in the general population. The 1^st^ tier mainly provided mental health education and helped people to mentally prepare themselves to deal with the crisis. The 2^nd^ tier of our psychological intervention (e.g., 24-hour hotlines and online interventions) mainly aimed to identify the target groups which were in need of intervention. Through our telephone hotlines and video psychotherapy platforms, we could quickly start the online consultations and establish problem feedback mechanisms in the 3^rd^ tier. In addition, the psychological rescue team conducted on-site crisis interventions for confirmed COVID-19 patients and/frontline medical staff in hospitals. The leading group positioned at the 4^th^ tier of the framework provided training and supervision during the entire process. Each team functioned as an independent unit to conduct psychological intervention through joint collaboration.

## Results

It was evident that our framework was proven feasible and successful in the provision of psychological assistance to those vulnerable subpopulations including the COVID-19 patients, suspected cases, close contacts, frontline medical staff, quarantined populations and the general public at large.

### Mental health education

Between 8^th^ of February and 26^th^ of March 2020, the live media group completed 45 episodes of live media programs about outbreak-associated psychological problems. Since the “Mind Filling Station” started broadcasting on the 8th of February, 147 internet derivatives have been created as audio/video-based programs. According to the data from SMR (one of the largest survey companies in China), our programs were broadcasted on four airwaves in Chengdu, with 11.72 million audience. Short audio/video programs and tweets were promoted by social media platforms including Weibo (mini blog posts), WeChat (chatting software) and TikTok (short video We Media) with nearly 30 million clicks and views.

### Hotline consultation

**Figure 4:**
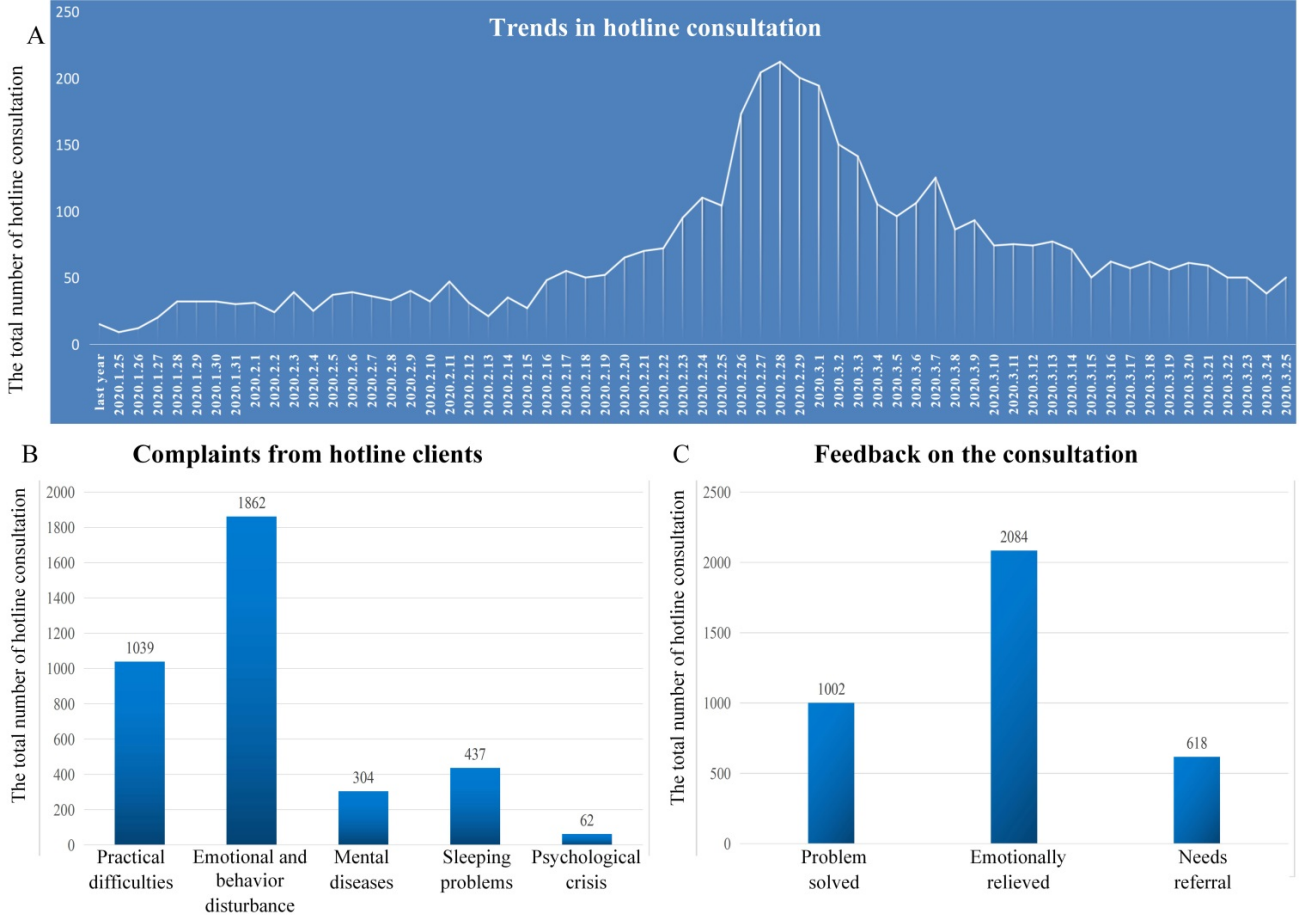
** A.** Trends in hotline consultation after the outbreak of the COVID-19 pandemic compared to the corresponding period of last year. **B.** Complaints from hotline clients. **C.** Feedback on the consultation.

Spanning across two months starting from the 26^th^ of January to the 26^th^ of March, there were 4,236 hotline consultations in total, with an average duration of 11.30 (± 8.48) minutes. Since the outbreak of the COVID-19 epidemic, the number of hotline consultations has risen rapidly compared to the corresponding period of last year (**Figure 4A**). By analyzing 3,704 valid hotline consultation data, we found that among the callers, women constituted the majority (57.54% were female, 42.46% were male). Regarding the type of complaints in consultations, 1039 cases were related to practical difficulties (28.05%), 1862 cases were associated with emotional and behavioral disturbances (50.27%), 304 cases involved the diagnosis and treatment of mental diseases (8.21%), 437 cases concerned their sleeping problems (12.80%), and 62 cases suffered from psychological crisis (1.67%) (**Figure 4B**). Regarding the feedback on our consultations, 27.05% of the clients stated that their problems were solved, 56.26% felt emotionally relieved, though practical difficulties still existed, and 16.69% had more complicated issues needing further intervention (**Figure 4C**). Our findings indicated that our COVID-19 model of psychological intervention achieved very satisfactory results and resolved most psychological crisis emerged during the COVID-19 pandemic.

### Online video psychotherapy

Between the 26th of January and the 26th of March, 233 patients received online video psychotherapy. A total of 84.97% of patients were redirected from the hotline consultation, and 15.03% were referred from COVID-19-designated hospitals and community-based observation spots.

### On-site crisis intervention

Between 26th of January to the 26th of March, we offered 70 one-on-one psychological interventions to 39 infected patients, one-third of which were treated with medication under the guidance of psychiatrists. We also conducted 5 psychological training sessions with frontline medical staff and received 98 visits from our target populations.

## Discussion

To the best of our knowledge, this is the first project in which “the integrated psychological intervention” was developed and implemented. The core idea is to integrate social media and internet technology into the process of intervention while incorporating the elements of preventive approach. Findings emerged have provided solid evidence that this type of intervention can achieve effective results in Chengdu, China.

Notably, the number of hotline consultations increased rapidly after the live media program was launched during the COVID-19 pandemic, possibly due to two main reasons: First, the public has increasing willingness to seek psychological assistance via a user-friendly media (e.g., hotlines and internet) 18. Through the new media-live broadcasts, we succeeded in raising public awareness of individuals' own psychological states 19. The combined model of media publicity and hotline/online video intervention increased the population's awareness of emotional disturbance after the outbreak of a severe public health crisis. This heightened awareness helped motivating individuals to obtain timely and effective psychological assistance.

According to the analysis of our hotline consultation data, emotional disturbance seemed to be the top priority problem, accounting for half of the consultations. Feedback from clients demonstrated that more than 50% of the callers felt their negative emotions, such as anxiety and depression, were relieved. Although we could not solve their practical problems in a timely fashion during the pandemic, we were able to help ease clients' emotional disturbances and avoid the flooding of negative emotions. Of particular note was that 16.69% of the clients reported more complicated issues due to early childhood trauma or excessive stress. Therefore, hotline consultation could not offer much help. Nonetheless, the hotline consultation was able to screen out the high-risk population and develop an effective intervention mechanism, which we considered very critical and important in the field of psychiatry and psychology. Through the referral mechanism, these clients could receive further assistance, such as online video intervention or on-site crisis intervention, to reduce the risk of psychological crisis events. When people realized their psychological difficulties, fear of exposure to the virus and the perceived stigma attached with their psychological problems might hinder their intention to seek help in mental health facilities 20, 21. Thus, remote psychological intervention played a crucial role in this pandemic situation.

Overall, three key highlights with regard to our integrated psychological intervention should be addressed. First, we paid more emphasis on individual's psychological status well before the crisis happened. Our intervention embedded with the use of multimedia enabled us to educate the public about mental health knowledge and offer them self-adjustment skills to improve their self-efficacy. Second, we offered hotline consultations to address the psychological issues that the public mostly concerned with, by using online video psychotherapy sessions to reduce their negative emotions to the minimal extent. Third, we provided timely on-site crisis intervention to the most at-risk group (COVID-19 patients, frontline medical staff) to prevent any psychological crisis events. With concerted effort, we had improved the overall public and mental health during the COVID-19 pandemic. We believed that this model can offer guidance for a systematic and effective psychological intervention program when there is any sudden outbreak of public health events in the near future.

However, there are some limitations on this model. Our methods of intervention, such as the hotline and online videos, were relatively dull, and the signal transmission might be delayed. We were unable to provide high-quality psychological interaction compared with face-to-face situations, which might reduce the effects of our intervention. Unfortunately, effective utilization of internet resources remained a major challenge in China 19. Due to occasional poor Wi-Fi reception, the software or program could not be connected with our remote system resulting in interruption during online psychological assessments. Thus, incorporation of artificial intelligence (AI) in devising a digital integrated platform with a human-oriented hotline service would be of great significance in the provision of psychological interventions in the near future.

## Conclusions

This newly developed COVID-19 Psychological Resilience Model has proven effective in this project. Our integrated psychological intervention program could be used as an expert reference guide to other regions in China or to other countries in the provision of effective psychological intervention which largely reduce negative mental health outcomes in a sudden outbreak of a public health emergency.

## Figures and Tables

**Table 1:** The 4-tiered structured psychological intervention program implementation

Tier	Psychological intervention program	Detailed implementation
The 1^st^ tier	Live media	The live media group launched a series of systematic and continuous mental health programs for the public called the “Mind Filling Station”, which was broadcasted on live from 8 pm to 9 pm every night through the Airing 9.14 App, twice a day.
The 2^nd^ tier	Hotline consultation	The consultation group provided 24/7 free consultation services by six psychological assistance hotlines (hotline number: 96008).
		Operators used a self-developed scale to collate data on documenting the source of information that callers were seeking, such as practical difficulties, sleeping problems, emotional or behavioral disturbances, diagnosis and treatment for mental diseases and psychological crisis states.
		Operators also solicited feedback from the clients immediately after the hotline consultation, using a three-option category: 1) “problem solved”, 2) “emotionally relieved”, or 3) “needs referral”.
The 3^rd^ tier	Online video intervention	This group utilized a remote video diagnosis and consultation system developed by the expert team of the Clinical Hospital of Chengdu Brain Science Institute, University of Electronic Science and Technology of China. Cases that were identified as 'complicated' or 'urgent' in the hotline consultation and those high-risk cases screened out by medical staff in COVID-19-designated hospitals or community-based observation spots were referred to online video intervention. Each session lasted for an average of 30 minutes or above.
	On-site crisis intervention	On-site crisis intervention was mainly provided for two groups of people 5, 22, 23. The first group included infected patients, suspected cases and quarantined cases who showed signs of psychological crisis. We trained the medical staff in COVID-19-designated hospitals and social workers in community-based observation spots in Chengdu to screen out cases in need of crisis intervention. We then assessed the mental health status of these cases. Appropriate intervention strategies were selected based on prior assessment with a follow-up plan.
		The second group was the frontline medical staff working in COVID-19-designated hospitals. We provided them with training based on the guidelines of Anticipate, Plan and Deter (APD) 24 so that they could effectively continue their job regardless.
The 4^th^ tier	Leading group	The leading group provided training and supervision during the entire process. They collected the feedback report of daily work of each team, hold weekly communication meeting so that they could coordinate and solve the existing problems and supervise the staff.
